# Association of thromboelastography profile with severity of liver cirrhosis and portal venous system thrombosis

**DOI:** 10.1186/s12876-021-01832-3

**Published:** 2021-06-07

**Authors:** Yanglan He, Shanshan Yuan, Xiaozhong Guo, Fangfang Yi, Xiangbo Xu, Yang An, Shixue Xu, Walter Ageno, Xingshun Qi

**Affiliations:** 1 Liver Cirrhosis Study Group, Department of Gastroenterology, General Hospital of Northern Theater Command (formerly General Hospital of Shenyang Military Area), No. 83 Wenhua Road, Shenyang, 110840 Liaoning Province China; 2 Department of Gastroenterology, Xi’an Central Hospital, Xi’an, 710003 China; 3 Postgraduate College, China Medical University, Shenyang, 110122 China; 4 Department of Medicine and Surgery, University of Insubria, Varese, Italy

**Keywords:** Thromboelastography, Hepatic, Cirrhosis, Thrombosis, Hypercoagulability

## Abstract

**Background and aim:**

Hemostasis profile is often complicated in liver cirrhosis. Thromboelastography is a global viscoelastic test recommended by the current practice guideline and consensus. This cross-sectional study aimed to evaluate the association of thromboelastography profile with severity of liver cirrhosis and presence of portal venous system thrombosis (PVST).

**Methods:**

Overall, 116 and 50 cirrhotic patients were included in the Shenyang and Xi’an cohorts, respectively. Thromboelastography parameters were compared between cirrhotic patients with Child–Pugh class A and B/C, those with and without decompensated events, and those with and without PVST. Hypercoagulability would be considered if at least two of the following thromboelastography parameters were met: shortened reactive time (R), shortened coagulation time (K), increased angle, and increased maximum amplitude (MA).

**Results:**

In the Shenyang cohort, 16 patients had shortened R, of whom seven (43.75%) had prolonged K and 11 (68.75%) decreased MA. In the Xi’an cohort, 24 patients had shortened R, of whom seven (29.17%) had prolonged K and 15 (62.50%) decreased MA. In the Shenyang cohort, the prevalence of hypercoagulability was not significantly different between cirrhotic patients with Child–Pugh class A and B/C (3.85% vs. 6.25%, *P* = 0.873), those with and without decompensated events (5.49% vs. 4.00%, *P* = 1.000), and those with and without PVST (4.17% vs. 5.88%, *P* = 1.000), which were similar to the results obtained in the Xi’an cohort.

**Conclusion:**

There is a high rate of discordance between R and other thromboelastography parameters. In addition, hypercoagulability may not be related to more advanced stage of liver cirrhosis or presence of PVST.

**Supplementary Information:**

The online version contains supplementary material available at 10.1186/s12876-021-01832-3.

## Background

Liver cirrhosis is a leading cause of morbidity and mortality worldwide [1], and is characterized by hemostatic imbalance [1–4]. Traditionally, it has been believed that liver cirrhosis carries a high risk of bleeding and hypocoagulation indicated by the conventional coagulation tests (CCTs), such as prolonged prothrombin time (PT), higher international normalized ratio (INR), and lower platelet (PLT) count. However, CCTs could not reflect real-time global hemostasis status of patients with cirrhosis [2, 4, 5]. By comparison, thromboelastography (TEG) is a novel global viscoelastic test, and the current guidelines and consensus have stated that TEG can evaluate the status of hemostasis more comprehensively [6, 7].

In this study, we aimed to describe the hemostasis status by TEG in cirrhotic patients and to explore the correlation of hemostatic changes with the severity of liver cirrhosis and presence of portal venous system thrombosis (PVST).

## Methods

### Population selection

This retrospective study included two cohorts (i.e., “*Shenyang cohort*” and “*Xi’an cohort*”). All adult patients with cirrhosis who were consecutively admitted to the Department of Gastroenterology of the General Hospital of Northern Theater Command and the Department of Gastroenterology of the Xi’an Central Hospital from July 1, 2018 to September 30, 2020 and underwent TEG test during their hospitalizations were potentially eligible for the study. Exclusion criteria were as follows: (1) a highly suspected or definite diagnosis of malignancy; (2) a known history of primary coagulopathy; (3) active infection at the time of TEG test; (4) a history of abdominal surgery [8], such as splenectomy [9], liver transplantation, transjugular intrahepatic portosystemic shunt, appendectomy, and cholecystectomy; (5) patients who received drugs with documented impact on coagulation status within 4 weeks prior to the TEG test; (6) a history of blood transfusion within 2 weeks prior to the TEG test; and (7) missing data of TEG test.

### Assessment of hemostasis status

CCTs mainly include PT, PLT, and fibrinogen (FIB). PT is used for evaluating the integrity of both extrinsic and common pathways of coagulation [10]. PLT and fibrinogen are used for evaluating the defects of the primary and secondary hemostasis.

TEG, a viscoelastic test, evaluates the global hemostasis state, mainly including the initiation of fibrin formation, rate of clot development, and maximum clot strength [11] (Additional file 1: Table S1). The TEG profile in the Shenyang cohort was assessed by the Thrombelastograph 5000 system (Haemoscope Corporation, USA), while the TEG profile in the Xi’an cohort was detected by the CFMSLEPU-8800 (Lepu Technology, China). In both Shenyang and Xi’an cohorts, the whole blood sample activated by citrated kaolin was added into a cup where the temperature remained 37 degrees Centigrade. A pin was placed in the sample when the cup was rotating. The sensor attached on the cup began to detect the viscoelastic changes of the sample once coagulation process was initiated. The TEG parameters mainly included reactive time (R), coagulation time (K), angle (α), maximum amplitude (MA). The hypercoagulability and hypocoagulability indicated by TEG parameters are shown in Additional file 1: Table S1.

### Diagnosis

A diagnosis of liver cirrhosis was mainly based on clinical, laboratory, and radiological examinations, and/or histological data, if necessary. Decompensated events included ascites, gastrointestinal bleeding, hepatic encephalopathy, and acute-on-chronic liver failure. The severity of liver cirrhosis was evaluated by Child–Pugh score. PVST was diagnosed according to the findings of contrast-enhanced computed tomography (CT)/magnetic resonance imaging (MRI) scans. PVST refers to thrombosis within portal vein trunk, intrahepatic portal vein, splenic vein, or mesenteric vein.

Hypercoagulability would be considered, if at least two of the following criteria were met: shortened R, shortened K, increased α, or increased MA as compared to the reference range.

### Statistical analysis

Qualitative data were described as frequency (percentage) and compared by χ^2^ or Fisher’s exact tests. Quantitative data were described by median (range) and compared by Mann–Whitney U test. The correlation between quantitative data was assessed by Spearman’s test. Statistical significance was defined as a *P* < 0.05. All the statistical data were calculated by IBM SPSS 22.0 (IBM Corp, Armonk, NY, USA). The violin plots demonstrating the distribution of CCTs and TEG and the scatter diagrams of R and other TEG parameters were drawn by the GraphPad Prism version 8.0 (GraphPad Software, Inc., La Jolla, California, USA).

## Results

### Patient characteristics

**Fig. 1:**
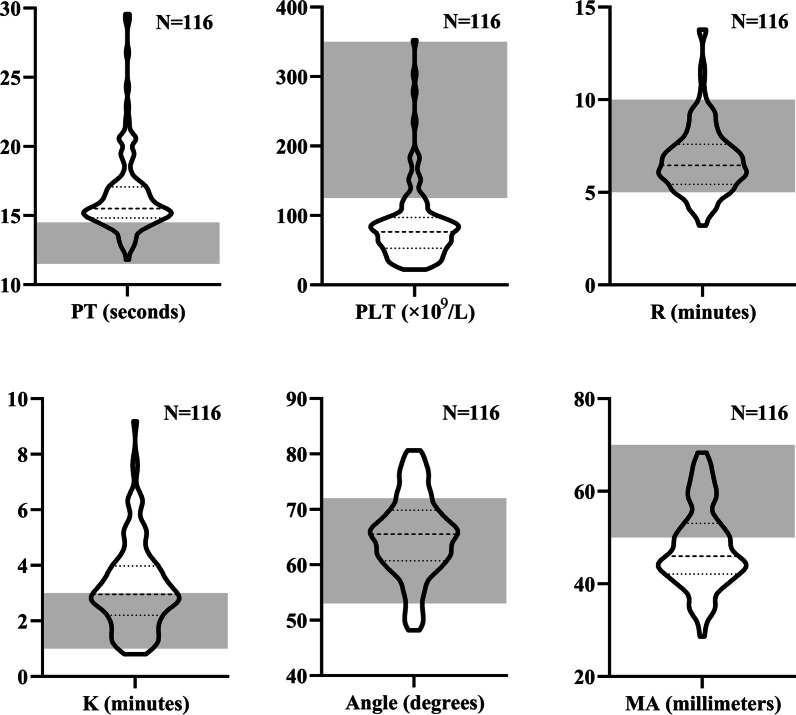
CCTs and TEG parameters in the Shenyang cohort. *Notes* Grey area represents the reference range of PT, PLT, and TEG parameters. N represents the number of patients undergoing PT, PLT, and TEG test

In the Shenyang cohort, 159 patients with cirrhosis underwent TEG test. Among them, 43 patients were excluded, 15 with definite or highly suspected diagnosis of malignancy, 2 with active infection, 16 with a history of abdominal surgery, 7 treated with drugs influencing coagulation status, 1 treated with fresh frozen plasma transfusion, and 2 with missing data. Finally, 116 patients were included, of whom 94 (81.03%) and 101 (87.06%) patients had abnormal PT and PLT, respectively (Fig. 1).

In the Xi’an cohort, 112 patients with cirrhosis underwent TEG test. Among them, 62 patients were excluded, 2 with definite or highly suspected diagnosis of malignancy, 3 with active infection, 34 with a history of abdominal surgery, 16 treated with drugs influencing coagulation status, and 7 with missing data. Finally, 50 patients were included, of whom 34 (69.39%) and 38 (76.00%) patients had abnormal PT and PLT, respectively (Additional file 2: Fig. S1).

### TEG profile

**Fig. 2:**
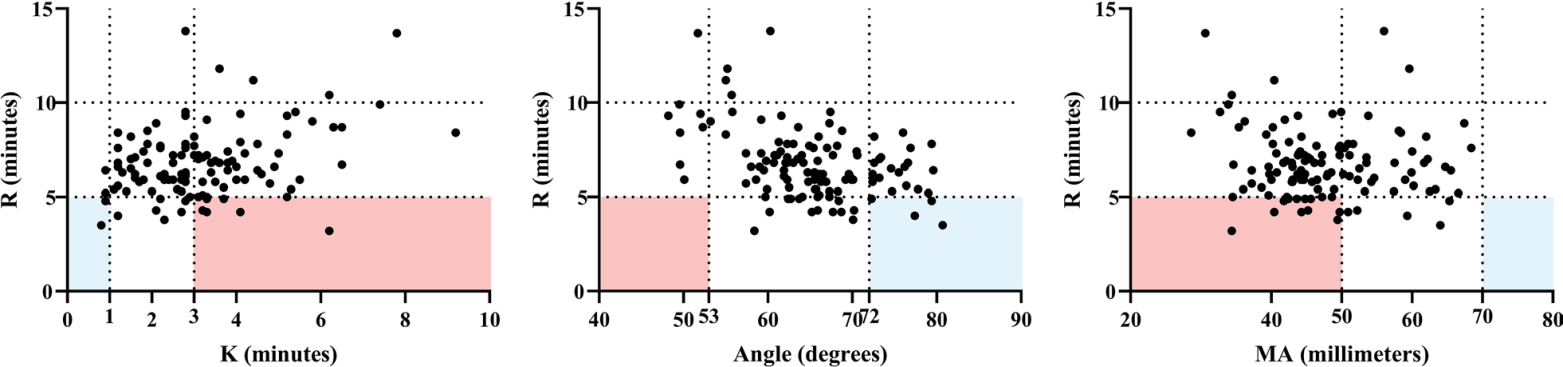
Consistency and discordance of R with other TEG parameters in the Shenyang cohort. *Notes* Blue area represents hypercoagulability indicated by both R and other TEG parameters. Red area represents hypercoagulability indicated by shorter R but hypocoagulability indicated by other TEG parameters. Dotted lines represent the reference ranges of TEG parameters

In the Shenyang cohort, 21 (18.10%), 59 (50.86%), 31 (26.72%), and 77 (66.38%) patients had abnormal R, K, α, and MA, respectively (Fig. 1). Sixteen (13.79%), four (3.45%), 23 (19.83%), and 0 (0%) patients had shortened R, shortened K, increased α, and increased MA as compared to the reference range, respectively. Among the 16 patients with shortened R, two (12.50%) had shortened K, four (25.00%) had increased α, and 0 (0.00%) had increased MA; by contrast, seven (43.75%) had prolonged K, 0 (0.00%) had decreased α, and 11 (68.75%) had decreased MA (Fig. 2). Six (5.17%) patients had hypercoagulability defined by TEG profile.

In the Xi’an cohort, 24 (48.00%), 21 (42.00%), eight (16.00%), and 34 (68.00%) patients had abnormal R, K, α, and MA, respectively (Additional file 2: Fig. S1). Twenty-four (48.00%), three (6.00%), six (12.00%), and two (4.00%) patients had shortened R, shortened K, increased α, and increased MA as compared to the reference range, respectively. Among the 24 patients with shortened R, three (12.50%) had shortened K, six (25.00%) had increased α, and two (8.33%) had increased MA; by contrast, seven (29.17%) had prolonged K, 0 (0.00%) had decreased α, and 15 (62.50%) had decreased MA (Additional file 3: Fig. S2). Six (12.00%) patients had hypercoagulability defined by TEG profile.

### Comparison between Child–Pugh class A and Child–Pugh class B/C cirrhosis

In the Shenyang cohort, there were 52 Child–Pugh class A and 64 Child–Pugh class B/C cirrhotic patients (Table 1). Compared to Child–Pugh class A cirrhotic patients, Child–Pugh class B/C cirrhotic patients had non-significantly shorter R, longer K, larger α, and lower MA. The proportions of hypocoagulability indicated by prolonged R, prolonged K, decreased α, and decreased MA were not significantly different between the two groups. The proportions of hypercoagulability indicated by shortened R, shortened K, increased α, and increased MA were not significantly different between them. The prevalence of hypercoagulability defined by TEG profile was not significantly different between the two groups (3.85% vs. 6.25%, *P* = 0.873).

**Table 1:** Difference of TEG profile between Child–Pugh class A and B/C cirrhosis in the Shenyang cohort

Variables	Child–Pugh class A	Child–Pugh class B/C	*P* value
	Median (range) or frequency (percentage)	Median (range) or frequency (percentage)	
*TEG profile*			
R (min)	6.80 (3.80–11.80)	6.20 (3.20–13.80)	0.119
Prolonged R	2/52 (3.85)	3/64 (4.69)	1.000
Shortened R	4/52 (7.69)	12/64 (18.75)	0.108
K (min)	2.90 (0.90–6.50)	2.95 (0.80–9.20)	0.923
Prolonged K	24/52 (46.15)	31/64 (48.44)	0.853
Shortened K	1/52 (1.92)	3/64 (4.69)	0.627
α (°)	64.15 (48.20–79.60)	65.80 (49.50–80.70)	0.355
Decreased α	4/52 (7.69)	4/64 (6.25)	1.000
Increased α	9/52 (17.31)	14/64 (21.88)	0.642
MA (mm)	47.15 (32.70–65.50)	45.00 (28.60–68.40)	0.445
Decreased MA	32/52 (61.54)	45/64 (70.31)	0.332
Increased MA	0/52 (0)	0/64 (0)	–
*Hypercoagulability*	2/52 (3.85)	4/64 (6.25)	0.873

R: Reaction time; K: Coagulation time; α: Angel; MA: Maximum Amplitude; min: minutes; °: degrees; mm: millimeters

In the Xi’an cohort, there were 15 Child–Pugh class A and 35 Child–Pugh class B/C cirrhotic patients (Additional file 4: Table S2). Compared to Child–Pugh class A cirrhotic patients, Child–Pugh class B/C cirrhotic patients had significantly lower MA, and non-significantly shorter R, longer K, and smaller α. The proportions of hypocoagulability indicated by prolonged R, prolonged K, decreased α, and decreased MA were not significantly different between the two groups. The proportions of hypercoagulability indicated by shortened R, shortened K, increased α, and increased MA were not significantly different between them. The prevalence of hypercoagulability defined by TEG profile was not significantly different between the two groups (6.67% vs. 14.29%, *P* = 0.776).

### Comparison between cirrhosis with and without decompensated events

In the Shenyang cohort, there were 91 and 25 cirrhotic patients with and without decompensated events, respectively (Table 2). Compared to those without, cirrhotic patients with decompensated events had significantly shorter R, and non-significantly shorter K, larger α, and lower MA. The proportions of hypocoagulability indicated by prolonged R, prolonged K, decreased α, and decreased MA were not significantly different between the two groups. The proportions of hypercoagulability indicated by shortened R, shortened K, increased α, and increased MA were not significantly different between them. The prevalence of hypercoagulability defined by TEG profile was not significantly different between the two groups (4.00% vs. 5.49%, *P* = 1.000).

**Table 2:** Difference of TEG profile between cirrhosis with and without decompensated events in the Shenyang cohort

Variables	Cirrhosis without decompensated events	Cirrhosis with decompensated events	*P* value
	Median (range) or frequency (percentage)	Median (range) or frequency (percentage)	
R (min)	7.10 (3.80–11.80)	6.20 (3.20–13.80)	*0.017*
Prolonged R	1/25 (4.00)	4/91 (4.40)	1.000
Shortened R	1/25 (4.00)	15/91 (16.48)	0.187
K (min)	3.00 (0.90–7.40)	2.90 (0.80–9.20)	0.882
Prolonged K	11/25 (44.00)	44/91 (48.35)	0.822
Shortened K	1/25 (4.00)	3/91 (3.30)	1.000
α (°)	64.20 (48.20–79.60)	65.50 (49.60–80.70)	0.539
Decreased α	4/25 (16.00)	4/91 (4.40)	0.065
Increased α	6/25 (24.00)	17/91 (18.68)	0.576
MA (mm)	49.40 (33.90–65.50)	45.50 (28.60–68.40)	0.452
Decreased MA	14/25 (56.00)	63/91 (69.23)	0.238
Increased MA	0/25 (0)	0/91 (0)	–
*Hypercoagulability*	1/25 (4.00)	5/91 (5.49)	1.000

*P* value in italics indicates statistical significance between the two groups

R: Reaction time; K: Coagulation time; α: Angel; MA: Maximum Amplitude; min: minutes; °: degrees; mm: millimeters

In the Xi’an cohort, there were 39 and 11 cirrhotic patients with and without decompensated events, respectively (Additional file 5: Table S3). Compared to those without, cirrhotic patients with decompensated events had significantly shorter R, longer K, and lower MA, and non-significantly smaller α. The proportions of hypocoagulability indicated by prolonged R, prolonged K, decreased α, and decreased MA were not significantly different between the two groups. Compared to those without, cirrhotic patients with decompensated events had a significantly higher proportion of hypercoagulability indicated by shortened R, but the proportions of hypercoagulability indicated by shortened K, increased α, and increased MA were not significantly different between the two groups. The prevalence of hypercoagulability defined by TEG profile was not significantly different between the two groups (0% vs. 15.38%, *P* = 0.317).

### Comparison between cirrhotic patients with and without PVST

In the Shenyang cohort, 75 patients underwent contrast-enhanced CT or MRI, of whom 24 had PVST and 51 did not have PVST (Table 3). PVST was asymptomatic in all of 24 patients. Compared to those without, cirrhotic patients with PVST had significantly lower MA, and non-significantly shorter R, longer K, and smaller α. The proportions of hypocoagulability indicated by prolonged R, prolonged K, decreased α, and decreased MA were not significantly different between the two groups. The proportions of hypercoagulability indicated by shortened R, shortened K, increased α, and increased MA were not significantly different between them. The prevalence of hypercoagulability defined by TEG profile was not significantly different between the two groups (5.88% vs. 4.17%, *P* = 1.000).

**Table 3:** Difference of TEG profile between cirrhosis with and without PVST in the Shenyang cohort

Variables	Cirrhosis without PVST	Cirrhosis with PVST	*P* value
	Median (range) or frequency (percentage)	Median (range) or frequency (percentage)	
*TEG profile*			
R (min)	6.80 (3.50–13.70)	6.30 (4.20–11.20)	0.436
Prolonged R	2/51 (3.92)	1/24 (4.17)	1.000
Shortened R	7/51 (13.73)	3/24 (12.50)	1.000
K (min)	2.80 (0.80–7.80)	3.60 (1.20–6.30)	0.064
Prolonged K	20/51 (39.22)	15/24 (62.50)	0.083
Shortened K	2/51 (3.92)	0/24 (0)	0.559
α (°)	65.90 (50.10–80.70)	63.55 (48.20–76.40)	0.121
Decreased α	4/51 (7.84)	1/24 (4.17)	0.667
Increased α	11/51 (21.57)	3/24 (12.50)	0.527
MA (mm)	48.50 (30.60–68.40)	43.75 (37.20–60.20)	*0.014*
Decreased MA	31/51 (60.78)	20/24 (83.33)	0.065
Increased MA	0/51 (0)	0/24 (0)	–
*Hypercoagulability*	3 (5.88)	1 (4.17)	1.000

*P* value in italics indicates statistical significance between the two groups

PVST: Portal Venous System Thrombosis; R: Reaction time; K: Coagulation time; α: Angel; MA: Maximum Amplitude; min: minutes; °: degrees; mm: millimeters

In the Xi’an cohort, 48 patients underwent contrast-enhanced CT or MRI, of whom 10 had PVST and 38 did not have PVST (Additional file 6: Table S4). PVST was asymptomatic in all of 10 patients. Compared to those without, cirrhotic patients with PVST had non-significantly shorter R, longer K, smaller α, and lower MA. Compared to those without, the cirrhotic patients with PVST had significantly higher proportion of hypocoagulability indicated by decreased α, but the proportions of hypocoagulability indicated by prolonged R, prolonged K, and decreased MA were not significantly different between the two groups. The proportions of hypercoagulability indicated by shortened R, shortened K, increased α, and increased MA were not significantly different between them. The prevalence of hypercoagulability defined by TEG profile was not significantly different between the two groups (13.16% vs. 0.00%, *P* = 0.569).

### Correlation between TEG and CCTs in cirrhosis

The correlation between TEG parameters and CCTs in cirrhosis is shown in Additional file 7: Table S5. In both Shenyang and Xi’an cohorts, K, α, and MA, but not R, significantly correlated with CCTs.

## Discussion

Patients with cirrhosis are often considered to be at risk for bleeding because of deteriorated or abnormal CCTs. Recent evidence demonstrated that elevated INR was not indicative of a higher risk for bleeding [12–14], as in such patients, the hemostatic status assessed by thrombin generation assays was rather normal [2, 4, 5] due to a simultaneous deficiency of both pro- and anti-coagulants [15]. Accordingly, current guideline and consensus do not recommend the use of conventional coagulation parameters for the assessment of coagulation status and bleeding risk in liver cirrhosis [6]. TEG can provide comprehensive information about function and amounts of pro-coagulants, anti-coagulants, and PLT as well as their interactions [16, 17]. In cirrhotic patients, TEG often shows normal global hemostasis, in spite of abnormal CCTs [18, 19]. Similarly, in our study, abnormal hemostatic status indicated by CCTs was more common than those indicated by TEG in both Shenyang and Xi’an cohorts.

In accordance with previous studies [17, 20], our current study showed a trend towards hypocoagulability, indicated by most of the TEG parameters, in patients with more advanced liver cirrhosis. However, it should be noted that R is often discordant with other TEG parameters. Both Shenyang and Xi’an cohorts demonstrated a lower MA, indicating the presence of hypocoagulability, in patients with decompensated events than those without; contrarily, a significantly shorter R, indicating the presence of hypercoagulability, in patients with decompensated events than those without. Such an opposite correlation is also observed among our individuals with shorter R but lower MA. This unexpected phenomenon could be explained by the fact that PLT and FIB were not closely associated with R [17, 21], but with K, α, and MA [17, 20]. Therefore, we could not arbitrarily define hypercoagulability by shortened R alone. Furthermore, our current study employed a new definition of hypercoagulability, which was different from previous study [17]. In details, in our study, hypercoagulability was diagnosed based on at least two of the following four TEG parameters: shortened R, shortened K, increased α, and increased MA. According to the diagnostic criteria, our study suggested that more advanced cirrhosis might not have a higher probability of hypercoagulability than less advanced cirrhosis.

A previous study by Zanetto et al. found that hypercoagulation indicated by higher MA was associated with PVST in cirrhotic patients with hepatocellular carcinoma [22]. Given that cancer itself could also trigger hypercoagulation, we could not extrapolate the association between TEG profile and PVST in non-malignant liver cirrhosis from the Zanetto’s findings. Huang et al. found a significantly shorter R in cirrhotic patients with non-tumoral PVST as compared to those without [23]. Notably, Huang et al. included only patients with gastroesophageal varices but we also included patients with other complications of liver cirrhosis. Though we had a large number of patients with shortened R, we did not find any significant difference in median R and proportion of shortened R between cirrhotic patients with and without PVST. Thus, our findings also suggested that hypercoagulability indicated by TEG profile might not be an important contributor to PVST formation in cirrhosis. This finding is similar to the findings of our previous meta-analysis that antithrombin, protein C, and protein S levels were not significantly different between patients with and without PVST after matching Child–Pugh class [24]. It has been widely recognized that the formation of PVST may be multifactorial in liver cirrhosis [25, 26]. After adjusting other thrombotic risk factors, such as antithrombin, protein C, lupus anticoagulant, cryoglobulins, and hyper-homocysteine, decreased portal vein velocity might be the most important risk factor for PVST formation [27].

Our study employed two cohorts to analyze TEG profile in liver cirrhosis, and the results in the Shenyang cohort were generally similar to those in the Xi’an cohort. However, there were several limitations in our study. First, TEG facilities and reference ranges are different at the two centers, in spite of the same reagents (citrated kaolin) and technical principles. Second, the characteristics of included patients were not the same between the two centers, which might contribute to the heterogeneity of TEG values obtained from the two cohorts. Third, our cirrhotic patients have asymptomatic PVST without acute abdomen. Thus, the correlation of TEG profile with the age of PVST could not be evaluated. Forth, we did not measure levels of coagulation factors to evaluate the association of shortened R with coagulation factors.

## Conclusion

TEG profile suggests a relatively normal hemostasis status in liver cirrhosis. However, there is a high rate of discordance between R and other TEG parameters. Hypercoagulability indicated by TEG parameters may not be related to more advanced stage of liver cirrhosis. Additionally, systemic hypercoagulability detected by TEG profile may not be a major contributor of PVST in liver cirrhosis.

## Supplementary Information

**Additional file 1: Table S1.** Hypercoagulability and hypocoagulability indicated by TEG parameters.

**Additional file 2: Fig. S1.** CCTs and TEG parameters in the Xi’an cohort. Notes: Grey area represents the reference range of PT, PLT, and TEG parameters. N represents the number of patients undergoing PT, PLT, and TEG test.

**Additional file 3: Fig. S2.** Consistency and discordance of R and TEG parameters in the Xi’an cohort. Notes: Blue area represents hypercoagulability indicated by both R and other TEG parameters. Red area represents hypercoagulability indicated by shorter R but hypocoagulability indicated by other TEG parameters. Dotted lines represent the reference ranges of TEG parameters.

**Additional file 4: Table S2.** Difference of TEG profile between Child-Pugh class A and B/C cirrhosis in the Xi'an cohort.

**Additional file 5: Table S3.** Difference of TEG profile between cirrhosis with and without decompensated events in the Xi'an cohort.

**Additional file 6: Table S4.** Difference of TEG profile between cirrhosis with and without PVST in the Xi'an cohort.

**Additional file 7: Table S5.** Correlation between TEG parameters and CCTs in two cohorts.

## Data Availability

The datasets generated during and/or analyzed during the current study are available from the corresponding author on reasonable request.

## Abbreviations

α: Angle
CCTs: Conventional coagulation tests
CT: Computed tomography
INR: International normalized ratio
K: Coagulation time
FIB: Fibrinogen
MA: Maximum amplitude
MRI: Magnetic resonance imaging
PLT: Platelet
PT: Prothrombin time
PVST: Portal venous system thrombosis
R: Reactive time
TEG: Thromboelastography
