# Supplementary material for: Association of thromboelastography profile with severity of liver cirrhosis and portal venous system thrombosis
Source: BMC Gastroenterol. 2021 Jun 7;21:253. doi: 10.1186/s12876-021-01832-3 (PMC8185912; doi:10.1186/s12876-021-01832-3)
Supplement: Supplementary file 1 — Additional file 1: Table S1. Hypercoagulability and hypocoagulability indicated by TEG parameters. [file 12876_2021_1832_MOESM1_ESM.docx]

| **Supplementary Table 1. Hypercoagulability and Hypocoagulability indicated by TEG parameters** | | | |
| --- | --- | --- | --- |
| **TEG parameters** | **Hemostasis phase indicated by TEG parameters** | **Hypocoagulability indicated by TEG parameters** | **Hypercoagulability indicated by TEG parameters** |
| R | Initiation of fibrin formation | Prolonged R compared to the upper limit of reference range | Shortened R compared to the lower limit of reference range |
| K | Rate of clot development | Prolonged K compared to the upper limit of reference range | Shortened K compared to the lower limit of reference range |
| α | Rate of clot development | Decreased α compared to the lower limit of reference range | Increased α compared to the upper limit of reference range |
| MA | Maximum clot strength | Decreased MA compared to the lower limit of reference range | Increased MA compared to the upper limit of reference range |
| **Abbreviations**: TEG: Thromboelastography; R: Reaction time; K: Coagulation time; α: Angle; MA: Maximum Amplitude. | | | |
