# Supplementary figures and images for: Association of thromboelastography profile with severity of liver cirrhosis and portal venous system thrombosis
Source: BMC Gastroenterol. 2021 Jun 7;21:253. doi: 10.1186/s12876-021-01832-3 (PMC8185912; doi:10.1186/s12876-021-01832-3)

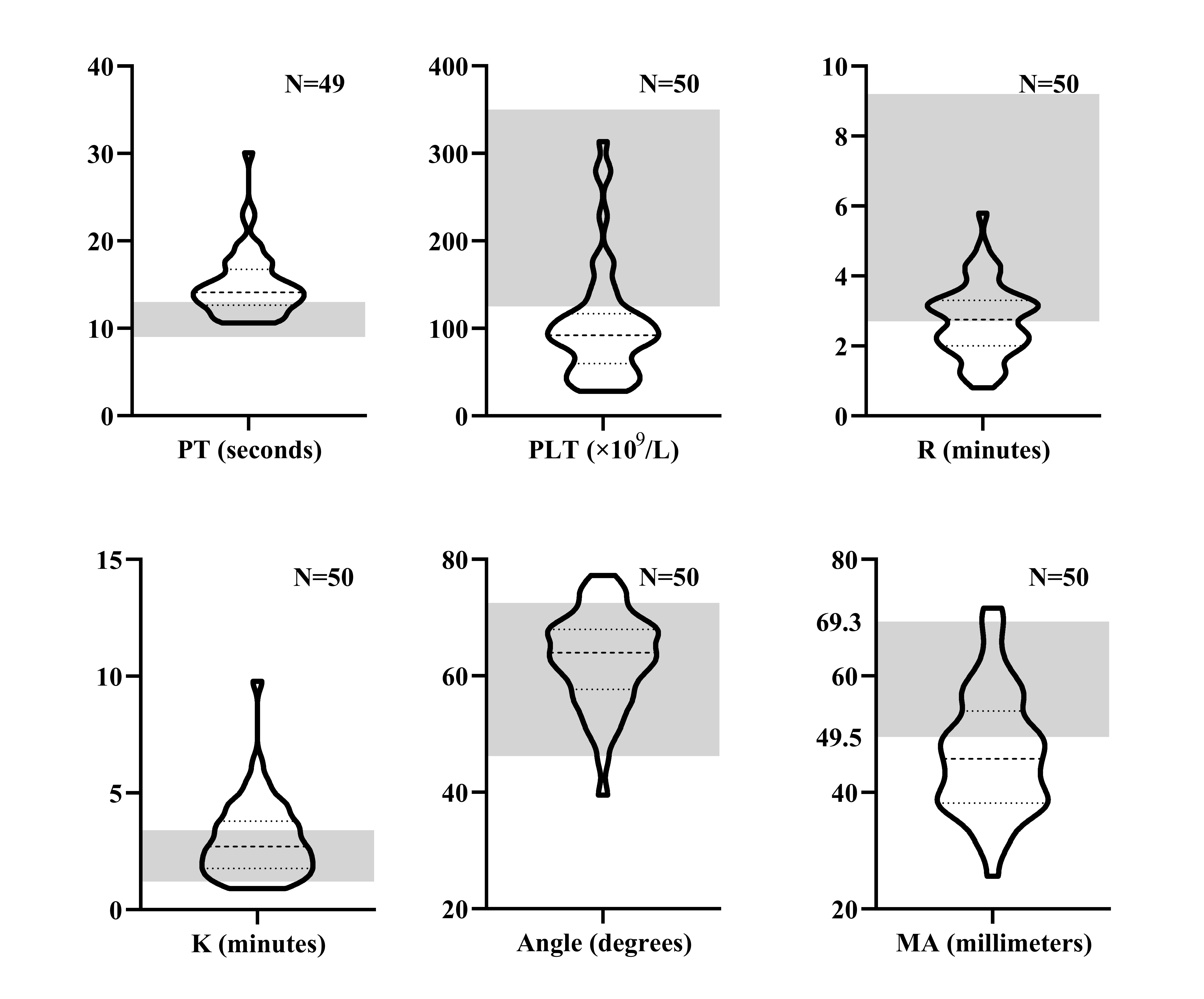

Supplement: Supplementary file 2 — Additional file 2: Fig. S1. CCTs and TEG parameters in the Xi’an cohort. Notes: Grey area represents the reference range of PT, PLT, and TEG parameters. N represents the number of patients undergoing PT, PLT, and TEG test. [file 12876_2021_1832_MOESM2_ESM.jpg]

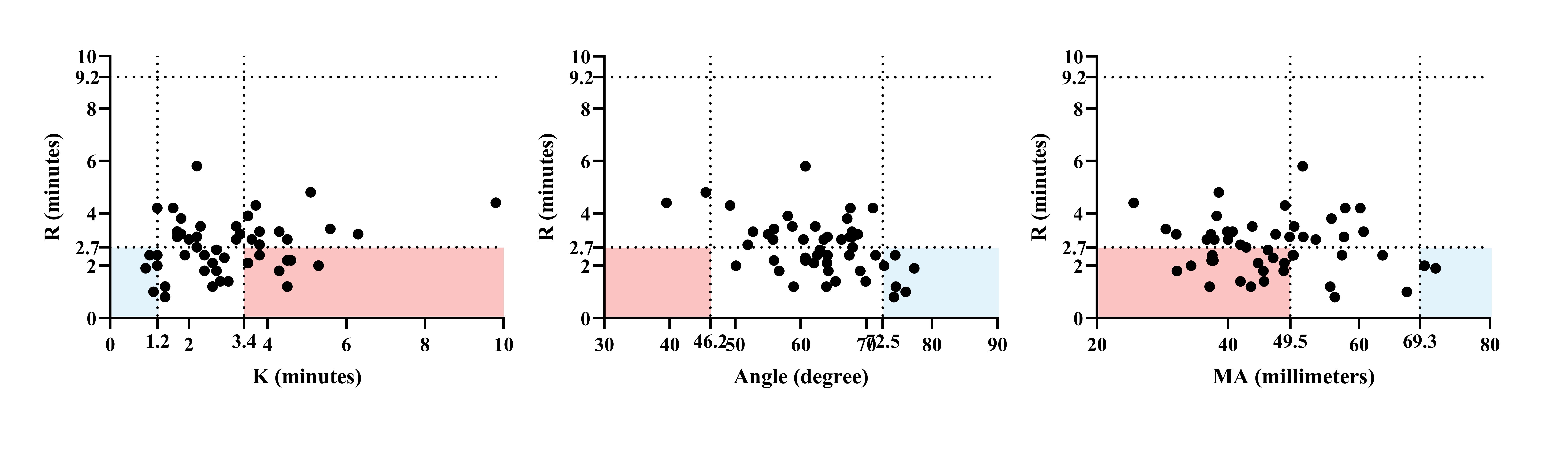

Supplement: Supplementary file 3 — Additional file 3: Fig. S2. Consistency and discordance of R and TEG parameters in the Xi’an cohort. Notes: Blue area represents hypercoagulability indicated by both R and other TEG parameters. Red area represents hypercoagulability indicated by shorter R but hypocoagulability indicated by other TEG parameters. Dotted lines represent the reference ranges of TEG parameters. [file 12876_2021_1832_MOESM3_ESM.jpg]
