# Supplementary material for: Association of thromboelastography profile with severity of liver cirrhosis and portal venous system thrombosis
Source: BMC Gastroenterol. 2021 Jun 7;21:253. doi: 10.1186/s12876-021-01832-3 (PMC8185912; doi:10.1186/s12876-021-01832-3)
Supplement: Supplementary file 4 — Additional file 4: Table S2. Difference of TEG profile between Child-Pugh class A and B/C cirrhosis in the Xi'an cohort. [file 12876_2021_1832_MOESM4_ESM.docx]

| **Supplementary Table 2. Difference of TEG profile between Child-Pugh class A and B/C cirrhosis in the Xi'an cohort** | | | | | |
| --- | --- | --- | --- | --- | --- |
| **Variables** | | **Child-Pugh class A** |  | **Child-Pugh class B/C** | **P value** |
|  |  | **Median (Range) or Frequency (Percentage)** |  | **Median (Range) or Frequency (Percentage)** |  |
| **TEG profile** | | | | | |
| R (minutes): | | 3.00 (1.80-4.20) |  | 2.60 (0.80-5.80) | 0.458 |
| - Prolonged R  - Shortened R | | 0/15 (0) 6/15 (40.0) |  | 0/35 (0)  18/35 (51.43) | - 0.545 |
| K (minutes): | | 2.20 (1.00-4.30) |  | 3.00 (0.90-9.80) | 0.086 |
| - Prolonged K  - Shortened K | | 3/15 (20.00) 1/15 (6.57) |  | 15/35 (42.86) 2/35 (5.71) | 0.199 1.000 |
| α (degree): | | 66.20 (52.70-74.40) |  | 62.50 (39.50-77.30) | 0.346 |
| - Decreased α  - Increased α | | 0/15 (0) 1/15 (6.67) |  | 2/35 (5.71) 5/35 (14.29) | 0.571 0.654 |
| MA (mm): | | 49.90 (37.90-63.60) |  | 43.50 (25.60-71.70) | ***0.039*** |
| - Decreased MA  - Increased MA | | 7/15 (46.67) 0/15 (0) |  | 25/35 (71.43) 2/35 (5.71) | 0.177 0.571 |
| **Hypercoagulability** | | 1/15 (6.67) |  | 5/35 (14.29) | 0.776 |
| **Abbreviations**: R: Reaction time; K: Coagulation time; α: Angel; MA: Maximum Amplitude. | | | | | |
