# Supplementary material for: Association of thromboelastography profile with severity of liver cirrhosis and portal venous system thrombosis
Source: BMC Gastroenterol. 2021 Jun 7;21:253. doi: 10.1186/s12876-021-01832-3 (PMC8185912; doi:10.1186/s12876-021-01832-3)
Supplement: Supplementary file 5 — Additional file 5: Table S3. Difference of TEG profile between cirrhosis with and without decompensated events in the Xi'an cohort. [file 12876_2021_1832_MOESM5_ESM.docx]

| **Supplementary Table 3. Difference of TEG profile between cirrhosis with and without decompensated events in the Xi'an cohort** | | | | |
| --- | --- | --- | --- | --- |
| **Variables** | **Cirrhosis without Decompensated Events** |  | **Cirrhosis with Decompensated Events** | **P value** |
|  | **Median (Range) or Frequency (Percentage)** |  | **Median (Range) or Frequency (Percentage)** |  |
| **TEG profile** | | | | |
| R (minutes): | 3.30 (2.10-5.80) |  | 2.40 (0.80-4.80) | ***0.016*** |
| - Prolonged R  - Shortened R | 0/11 (0) 2/11 (18.18) |  | 0/39 (0) 22/39 (56.41) | ***- 0.040*** |
| K (minutes): | 1.80 (1.20-3.80) |  | 3.00 (0.90-9.80) | ***0.036*** |
| - Prolonged K  - Shortened K | 1/11 (9.09) 0/11 (0) |  | 17/39 (43.59) 3/39 (7.69) | 0.072 0.584 |
| α (degree): | 67.50 (52.80-71.40) |  | 62.90 (39.50-77.30) | 0.303 |
| - Decreased α  - Increased α | 0/11 (0) 0/11 (0) |  | 2/39 (5.13) 6/39 (15.38) | 1.000 0.317 |
| MA (mm): | 51.40 (39.90-60.70) |  | 44.60 (25.60-71.70) | ***0.043*** |
| - Decreased MA  - Increased MA | 5/11 (45.45) 0/15 (0) |  | 27/39 (69.23) 2/39 (5.13) | 0.172 1.000 |
| **Hypercoagulability** | 0 (0) |  | 6 (15.38) | 0.317 |
| **Abbreviations**: R: Reaction time; K: Coagulation time; α: Angel; MA: Maximum Amplitude. | | | | |
