# Supplementary material for: Association of thromboelastography profile with severity of liver cirrhosis and portal venous system thrombosis
Source: BMC Gastroenterol. 2021 Jun 7;21:253. doi: 10.1186/s12876-021-01832-3 (PMC8185912; doi:10.1186/s12876-021-01832-3)
Supplement: Supplementary file 6 — Additional file 6: Table S4. Difference of TEG profile between cirrhosis with and without PVST in the Xi'an cohort. [file 12876_2021_1832_MOESM6_ESM.docx]

| **Supplementary Table 4. Difference of TEG profile between cirrhosis with and without PVST in the Xi'an cohort** | | | | |
| --- | --- | --- | --- | --- |
| **Variables** | **Cirrhosis without PVST** |  | **Cirrhosis with PVST** | **P value** |
|  | **Median (Range) or Frequency (Percentage)** |  | **Median (Range) or Frequency (Percentage)** |  |
| **TEG profile** | | | | |
| R (minutes): | 2.90 (0.80-5.80) |  | 2.35 (1.40-4.80) | 0.629 |
| - Prolonged R  - Shortened R | 0/38 (0) 17/38 (44.74) |  | 0/10 (0) 6/10 (60.00) | - 0.487 |
| K (minutes): | 2.60 (0.90-6.30) |  | 3.60 (1.70-9.80) | 0.080 |
| - Prolonged K  - Shortened K | 13/38 (34.21) 2/38 (5.26) |  | 5/10 (50.00) 0/10 (0) | 0.468 1.000 |
| α (degree): | 64.05 (49.20-77.30) |  | 58.70 (39.50-69.90) | 0.200 |
| - Decreased α  - Increased α | 0/38 (0) 5/38 (13.16) |  | 2/10 (20.00) 0/10 (0) | ***0.040*** 0.347 |
| MA (mm): | 46.70 (30.50-71.70) |  | 40.25 (25.60-60.70) | 0.091 |
| - Decreased MA  - Increased MA | 23/38 (60.53) 0/38 (0) |  | 8/10 (80.00) 0/10 (0) | 0.298 1.000 |
| **Hypercoagulability** | 5 (13.16) |  | 0 (0.00) | 0.569 |
| **Abbreviations**: PVST: Portal Vein System Thrombosis; R: Reaction time; K: Coagulation time; α: Angel; MA: Maximum Amplitude. | | | | |
