# Supplementary material for: Association of thromboelastography profile with severity of liver cirrhosis and portal venous system thrombosis
Source: BMC Gastroenterol. 2021 Jun 7;21:253. doi: 10.1186/s12876-021-01832-3 (PMC8185912; doi:10.1186/s12876-021-01832-3)
Supplement: Supplementary file 7 — Additional file 7: Table S5. Correlation between TEG parameters and CCTs in two cohorts. [file 12876_2021_1832_MOESM7_ESM.docx]

| **Supplementary Table 5. Correlation between TEG parameters and CCTs in two cohorts** | | | | | | | | | | | |
| --- | --- | --- | --- | --- | --- | --- | --- | --- | --- | --- | --- |
|  | R | |  | K | |  | α | |  | MA | |
|  | r | P value |  | r | P value |  | r | P value |  | r | P value |
| PT in Shenyang | 0.039 | 0.676 |  | 0.420 | ***<0.001*** |  | -0.287 | ***0.002*** |  | -0.470 | ***<0.001*** |
| PT in Xi'an | 0.082 | 0.574 |  | 0.547 | ***<0.001*** |  | -0.388 | ***0.006*** |  | -0.619 | ***<0.001*** |
| PLT in Shenyang | -0.098 | 0.297 |  | -0.688 | ***<0.001*** |  | 0.604 | ***<0.001*** |  | 0.743 | ***<0.001*** |
| PLT in Xi'an | -0.081 | 0.578 |  | -0.645 | ***<0.001*** |  | 0.437 | ***0.002*** |  | 0.648 | ***<0.001*** |
| FIB in Shenyang | 0.144 | 0.124 |  | -0.620 | ***<0.001*** |  | 0.456 | ***<0.001*** |  | 0.735 | ***<0.001*** |
| FIB in Xi'an | -0.019 | 0.895 |  | -0.549 | ***<0.001*** |  | 0.369 | ***0.008*** |  | 0.606 | ***<0.001*** |
| **Abbreviations**: TEG: Thromboelastography; CCTs: Conventional Coagulation Tests; PT: Prothrombin time; PLT: Platelet count; FIB: Fibrinogen; r: Correlation Coefficient; R: Reaction time; K: Coagulation time; α: Angel; MA: Maximum Amplitude. | | | | | | | | | | | |
